# Etiological Beliefs, Treatments, Stigmatizing Attitudes toward Schizophrenia. What Do Italians and Israelis Think?

**DOI:** 10.3389/fpsyg.2017.02289

**Published:** 2018-01-09

**Authors:** Stefania Mannarini, Marilisa Boffo, Alessandro Rossi, Laura Balottin

**Affiliations:** ^1^Interdepartmental Center for Family Research, Department of Philosophy, Sociology, Education, and Applied Psychology, Section of Applied Psychology, University of Padua, Padua, Italy; ^2^Addiction, Development and Psychopathology (ADAPT) Lab, Department of Psychology, University of Amsterdam, Amsterdam, Netherlands

**Keywords:** cross-cultures, schizophrenia, causal beliefs, dangerousness, closeness, avoidance, treatment, latent class analysis

## Abstract

**Background:** Although scientific research on the etiology of mental disorders has improved the knowledge of biogenetic and psychosocial aspects related to the onset of mental illness, stigmatizing attitudes and behaviors are still very prevalent and pose a significant social problem.

**Aim:** The aim of this study was to deepen the knowledge of how attitudes toward people with mental illness are affected by specific personal beliefs and characteristics, such as culture and religion of the perceiver. More precisely, the main purpose is the definition of a structure of variables, namely perceived dangerousness, social closeness, and avoidance of the ill person, together with the beliefs about the best treatment to be undertaken and the sick person’ gender, capable of describing the complexity of the stigma construct in particular as far as schizophrenia is concerned.

**Method:** The study involved 305 university students, 183 from the University of Padua, Italy, and 122 from the University of Haifa, Israel. For the analyses, a latent class analysis (LCA) approach was chosen to identify a latent categorical structure accounting for the covariance between the observed variables. Such a latent structure was expected to be moderated by cultural background (Italy versus Israel) and religious beliefs, whereas causal beliefs, recommended treatment, dangerousness, social closeness, and public avoidance were the manifest variables, namely the observed indicators of the latent variable.

**Results:** Two sets of results were obtained. First, the relevance of the manifest variables as indicators of the hypothesized latent variable was highlighted. Second, a two-latent-class categorical dimension represented by prejudicial attitudes, causal beliefs, and treatments concerning schizophrenia was found. Specifically, the differential effects of the two cultures and the religious beliefs on the latent structure and their relations highlighted the relevance of the observed variables as indicators of the expected latent variable.

**Conclusion:** The present study contributes to the improvement of the understanding of how attitudes toward people with mental illness are affected by specific personal beliefs and characteristics of the perceiver. The definition of a structure of variables capable of describing the complexity of the stigma construct in particular as far as schizophrenia is concerned was achieved from a cross-cultural perspective.

## Introduction

The stigmatization of mentally ill individuals and the endorsement of stigmatizing beliefs and attitudes about the causal underpinnings of mental disorders are well documented in the scientific literature (Crisp et al., 2000; Read and Harré, 2001; Phelan et al., 2006; Livingston and Boyd, 2010; Angermeyer et al., 2011; Corrigan et al., 2012; Schomerus et al., 2012; Mannarini and Boffo, 2015). Although scientific research on the etiology of mental disorders has improved the knowledge of both biogenetic and psychosocial factors related to the onset of mental illness (Coyne and Downey, 1991; Kendler and Prescott, 2006), stigmatizing attitudes and behaviors as avoidance, negative stereotypes, adverse treatment in interactions, and several forms of discrimination are still present and pose a significant social problem (e.g., Svensson and Hansson, 2016). Stigmatization can be a serious impediment in everyday interpersonal relations, by also affecting treatment seeking and quality of life (Hinshaw, 2007; Livingston and Boyd, 2010). Studies concerning the relationship between etiological beliefs and stigmatizing attitudes toward mentally ill people have been carried out in different countries. Among different mental disorders stigma is studied in particular in relation to schizophrenia (Mehta and Farina, 1997; Read and Harré, 2001; Walker and Read, 2002; Angermeyer and Matschinger, 2005; Rüsch et al., 2005; Jorm and Griffiths, 2008; Lincoln et al., 2008; Rusch et al., 2009; Cook and Wang, 2011). Among others, a cross-cultural study conducted in Germany, Russia, and Mongolia has analyzed the relationship between mental disorders, causal beliefs, and social distance (Dietrich et al., 2004). Despite the different cultural backgrounds, the results have shown similar trends concerning the attribution of depression and schizophrenia to psychological causes, such as acute stress due to life events and in some cases to social and political events typical of a specific culture. Further, the importance of cross-cultural results suggested taking into consideration also the religious faith (regardless of the specific type of religion) as a variable which might interact with causal beliefs and attitudes toward mental disorders. Wesselmann and Graziano (2010) indeed argue that even if religious beliefs affect people’s behaviors toward others, only a few studies have examined religious stereotypes about people with mental illness (Wesselmann and Graziano, 2010). White et al. (2003) have identified three such religious stereotypes: attribution of mental disorder to demonic possession; mental illness as punishment from god for moral weakness; and mental illness as a consequence of insufficient faith in god or ineffective prays for healing. Wesselmann et al. (2015) in their study revealed a tendency of individuals who perceive themselves as religious to endorse biogenetic causes and consequently to support medical treatments in addition to spiritual treatments for mental illnesses. Stigma seems to be multifaceted, composed by several processes in terms of the origin of the mental illness, which interact and converge to attitudes or a behaviors, which are often characterized by discrimination, feeling unsafe, and avoidance (Jones et al., 1984; Feldman and Crandall, 2007). The next important problem involves people suffering of different mental problems, being probably stigmatized for motives connected with the specific diagnostic category (which implies different psychosocial-biogenetic causal attributions) they have been classified in Mannarini and Boffo (2014a, 2015).

The aim of this study was to deepen the understanding of how attitudes toward people with mental illness are affected by specific personal beliefs and characteristics of the perceiver. In particular, mental illness etiological beliefs were studied in relation to how people perceive dangerousness, social closeness, avoidance of the ill person, together with the beliefs about the best treatment to be undertaken and the sick person’ gender. The purpose was the delineation of a structure composed by the mentioned variables capable of describing the complexity of the stigma construct in particular as far as schizophrenia is concerned. The study was carried out in Italy and Israel in order to investigate whether different cultural backgrounds might influence people’s opinions and attitudes toward schizophrenia. Demographic variables of the participants such as age, gender, religious views, and past experience with mental illness were also taken into account. Among other mental disorders, schizophrenia was selected for two main reasons. First, schizophrenia is familiar among the general population. Second, because it was extensively investigated, there would be a well-known context for the interpretation of the findings of this study. Concerning different cultures, Italy and Israel are compared in this study mainly because in both countries the problem of stigma toward mental illness is taken into deep consideration, mediated through cultural and social factors typical of those societies (Tal et al., 2007; Munizza et al., 2013; Mannarini and Boffo, 2015). Differences between responses of Italian and Israeli participants were expected, due to daily social situations that people experience in their different cultures (Craparo et al., 2013; Gelkopf et al., 2013; Mannarini et al., 2017).

Hypotheses were formulated in relation to the structure which might characterize the variables interactions. To this purpose, in line with previous studies (Read and Harré, 2001; Mannarini and Boffo, 2013, 2015), since biogenetic and psychosocial mental illness causal attributions were considered as the extremes of a continuum, a relation between causal models of mental illness, perception of dangerousness, social closeness to sick people, and perception of a tendency to avoid sick people in the public opinion was expected (Dietrich et al., 2006; Angermeyer et al., 2011; Kvaale et al., 2013; Lee et al., 2014). In particular, previous studies revealed that substance addiction and schizophrenia were considered to be particularly dangerous in public opinion (Schomerus et al., 2011). In addition, following these premises, an expected link between beliefs regarding the recommended treatment and etiological beliefs was hypothesized to be part of the latent structure (McLellan et al., 2000). According to these expectations, multi-categorical interactions were hypothesized. Mental illness, etiological beliefs, perceived dangerousness, social closeness to sick people, avoidance, patient’s gender, and recommended treatments should be combined in a latent structure; such a structure should then include relevant aspects of stigma toward schizophrenia and should investigate as well the differential effect played by specific attributes of the respondents, in particular the cultures they belong to. In order to model this structure at a latent level, a one-factor latent class analysis (LCA) (Hagenaars and McCutcheon, 2006) model was hypothesized, with two external variables, (i.e., country of the respondent and religious belief) and six manifest variables (gender of the patient, causal beliefs, perceived dangerousness, social closeness, avoidance, and recommended treatment) which should be associated with each other. In particular, as perceived in the public opinion, perceived dangerousness should be linked with low social closeness and high avoidance, and biogenetic causal beliefs should be related to medical treatments rather than to psychotherapy. Participants’ cultural background should act as a mediator within the hypothesized interrelations (Marie and Miles, 2008; Angermeyer et al., 2011; Schomerus et al., 2012; Kvaale et al., 2013; Lee et al., 2014).

## Materials and Methods

### Participants and Procedure

The study involved 305 university students, 183 from the University of Padua, Italy, and 122 from the University of Haifa, Israel. Italian students’ mean age was 24.38 (*SD* = 5.2), 65% were female, 16% professed a religion, and 63% had some previous direct interaction with mentally ill people. The Israeli students’ mean age was 24.35 (*SD* = 4.4), 58% were female, 17% were religious, and 69% had some previous direct experience with mentally ill people. Most of the participants were studying social science courses (i.e., psychology) both in Italy and in Israel, while only 25% were studying scientific courses such as engineering.

4.5% of the total 319 students contacted refused to take part in the research. The study was presented during regular lectures of a university course as an example of how to conduct scientific research in the field of mental health and treatment. Students who refused to participate in the research did not have any downside in fulfilling the course requirements. The project was approved by (A) the Ethics Committee for Psychological Research of the University of Padua (Protocol number 1734) and by (B) the Haifa University (according to the regulation code of the Haifa University Ethics Review Board). Participants’ informed consent for confidential data treatment was part of the questionnaire package.. Written informed consent was obtained from all participants after the procedure had been fully explained and data anonymity for research use only was guaranteed by assigning to participants an identification number before starting the study. The participants were free to withdraw from the study at any time. Collected data were then aggregated and analyzed at group level. In the questionnaire the meaning of professing a religion was clearly defined as believing in a religion and in its creed, regardless of how many times the participant attends celebrations or visits churches or temples. Having contacts with mentally ill people was declined as a personal experience with a mentally ill person, however short or occasional.

### Instruments

#### Vignettes

Most of the questionnaires validated for the Italian context assessing attitudes and beliefs toward mental disorders, such as the *Community Attitudes to the Mentally Ill* (Buizza et al., 2005), the *Questionnaire on the Opinions about Mental Illness* (Magliano et al., 1999), the *Attribution Questionnaire-27* (Corrigan et al., 2003; Pingani et al., 2012) the *Mental Disorders Causal Beliefs scale*, (Mannarini and Boffo, 2013) and the *Mental Disorder Therapy Relationship scale* (Mannarini et al., 2013), refer to mental illness as a broad category without distinguishing any specific diagnostic category. However, each of these instruments does not consider the multifaceted structure of mental illness stigma, primarily focusing only on partial aspects such as beliefs and/or attitudes and treatment. Therefore, a vignette approach was used to specifically examine participants’ attitudes toward schizophrenia. The vignette approach also allowed simultaneous evaluation of causal beliefs, opinions about the recommended treatments, perceived dangerousness, desire for social distance, and avoidance, specifically related to schizophrenia. The participants received a vignette describing an unlabeled clinical case with a brief sketch about a university student having typical symptoms and problems of schizophrenia. The vignette approach was similar to Mannarini and Boffo (2015) and consistent with previous studies using a similar methodology (Link et al., 1999; Angermeyer and Matschinger, 2005; Jorm et al., 2006; Mannarini and Boffo, 2015). The problems and symptoms described followed the DSM-IV-TR (American Psychiatric Association, 2000) diagnostic criteria for schizophrenic disorder, without mentioning it in the text. In addition, the vignette protagonist was counterbalanced by sex across participants.

##### Causal beliefs

The participants indicated their agreement on ten possible etiologies of the problems of the person described in the vignette, where five items were related to biogenetic causes (i.e., genetic predisposition, physical disease, and health weakness) and the other five items referred to psychosocial causes (i.e., traumatic experiences during childhood, condition of socialization, and deprived family environment). Cronbach’s alpha for the scale was equal to 0.65 for the Italian version and 0.60 for the Israeli.

##### Recommended treatments

The participants were asked to evaluate the suitability of seven possible treatment approaches to alleviate the problems and symptoms of the vignettes protagonist. Three items suggested psychiatric treatments such as medication, hospitalization, and surgical operation, and four items covered more psychological therapeutic approaches, including psychotherapy, family therapy, and group therapy. Cronbach’s alpha for this scale was equal to 0.61 in the Italian version and 0.63 for the Israeli.

##### Social closeness

Desire for social closeness to the mentally ill person was assessed with five items that investigated the participant’s willingness to be in a relationship with the person described in the vignette. The relationship varied in the degree of closeness, ranging from romantic partner, close friend, colleague, to neighbors and acquaintances. Cronbach’s alpha for this scale was equal to 0.79 for the Italian version and 0.71 for the Israeli one.

##### Perceived dangerousness

Perceived dangerousness was evaluated with five items assessing the possibility that the vignette’s protagonist would manifest violent behaviors toward others, uncontrolled behaviors, violence behaviors. Cronbach’s alpha for this scale was equal to 0.80 for Italy and 0.75 for Israel.

##### Avoidance

The tendency of public opinion to avoid any involvement with that person was evaluated with four items asking an opinion about what people would generally do when involved with a mentally ill person.

All items were rated on an agreement Likert-type scale ranging from 1 (completely disagree) to 4 (completely agree). In order to perform the LCA analysis, the items of both the psychosocial causal beliefs and the psychological treatment approach were score-reversed. Higher scores for social distance, dangerousness, and avoidance indicated high degrees of vicinity, dangerousness, and avoidance, respectively. The questionnaires were administered in Italian and in Hebrew to the Italian and to the Israeli students, respectively. A back translation was used in order to guarantee the face and content validity between the two languages.

### Data Analysis

An LCA (Hagenaars and McCutcheon, 2006) approach was chosen to identify a latent categorical construct accounting for the covariance between the observed variables. LCA models the latent factor to be composed of a number of classes, which describe the presence or absence of the characteristics of the latent variable. LCA has been extensively used in the psychological and medical literature to model the underlying structure of variables of interest, with the goal of identify “profiles” of people based on their probability of falling into one class or another (Garrett et al., 2002; Romano et al., 2004; Campbell et al., 2009; Bornovalova et al., 2010; Tsai et al., 2014; Mannarini et al., 2016; Balottin et al., 2017). In our study, the latent factor was supposed to be composed of a number of classes, each one class conceptually representing a ‘profile’ of beliefs and attitudes toward schizophrenia in terms of the type of etiological beliefs, recommended treatment approaches, perceived dangerousness of people suffering from schizophrenia, desired social closeness to them, and general avoidance. Two classes representing the overall pattern of reactions toward schizophrenia were hypothesized (Mannarini and Boffo, 2013): one was mainly represented by the endorsement of psychosocial causal beliefs, psychological treatments, low dangerousness, high degree of closeness to the mentally ill person, and perception of no avoidance in the public opinion, whereas the other was represented by a greater endorsement of biogenetic causal beliefs, medical treatments, high dangerousness, low desire of closeness to the mentally ill person, and perception of avoidance in the public opinion. Such a latent structure was expected to be moderated by cultural background (Italy versus Israel) and religious beliefs, which were added in the model as covariates of the latent factor, whereas causal beliefs, recommended treatment, dangerousness, social closeness, and avoidance were the manifest variables, namely the observed indicators of the latent variable. The gender of the vignette protagonist was also added to the model.

Consistent with Blanton and Jaccard’s (2006) recommendations on the treatment of continuous variable as discrete, and based on previous studies (Mannarini and Boffo, 2013; Mannarini et al., 2016, 2017), the distributions of participants’ total scores to the vignettes scales were discretized using the percentile ranks to define three score categories for each variable: Category 1 for both causal beliefs and recommended treatment variables identified the clear endorsement of psychosocial beliefs/psychological treatment approaches (scores falling below the 33rd percentile), Category 2 referred to bio-psychosocial etiological beliefs/integrated treatment approaches (scores between the 33rd and 66th percentiles), and Category 3 to a stronger preference for biogenetic etiology/medical treatment approaches (scores above the 66th percentile). A similar procedure was applied to perceived dangerousness, closeness, and avoidance: Category 1 represented low dangerousness, low desire for closeness, and low perception of avoidance (scores below the 33rd percentile), Category 2 identified medium dangerousness, medium closeness, and medium avoidance (scores between the 33rd and 66th percentiles), Category 3 referred to high dangerousness, high closeness, and high perception of avoidance (scores above the 66th percentile).

To identify the best model fitting the data, the goodness of fit of the two-class model was compared with those of a one-class and a three-class model by examining the Akaike Information Criterion (AIC) (Akaike, 1987) and the Bayesian Information Criterion (BIC) (Schwarz, 1978), which decrease as the model fit improves. Considering the argumentation of Nylund et al. (2007) about the insufficiency of the AIC and BIC criteria in selecting the right number of classes also, the L^2^ likelihood ratio was taken into account.

The LCA analysis was performed with the LEM software (Vermunt, 1997).

## Results

### The Latent Classes

The LCA identified the two-latent-class model as fitting the data satisfactorily (*L*^2^ = 1013.69, *df* = 1877, *p* > 0.90; AIC = -2740.31, BIC = -9723.34). As shown in **Table 1**, the probability parameter estimates for each latent class revealed their unique characteristics.

**Table 1:** Latent class analysis (LCA) probability values for the external variables and the manifest variables for each latent class.

Latent class		Class 1 (0.26)					Class 2 (0.74)				
External variable		Country			Religious beliefs		Country			Religious beliefs	
		Italy	Israel		No	Yes	Italy	Israel		No	Yes
		0.13	0.87		0.39	0.61	0.74	0.26		0.45	0.55
**Manifest variable**				**Class Prob.**					**Class Prob.**		
Patient’s gender	M			0.46					0.50		
	F			**0.54**					0.50		
Causal belief	1			0.32					0.35		
	2			**0.50**					0.30		
	3			0.18					0.35		
Treatment	1			0.23					**0.52**		
	2			0.35					0.26		
	3			**0.41**					0.21		
Closeness	1			0.13					**0.53**		
	2			**0.41**					0.24		
	3			**0.46**					0.23		
Dangerousness	1			0.12					**0.58**		
	2			0.11					0.25		
	3			**0.78**					0.17		
Avoidance	1			**0.77**					0.31		
	2			0.12					**0.40**		
	3			0.11					0.29		

*Causal belief: 1 = Psychological, 2 = Bio-Psychological, 3 = Biological; Treatment: 1 = Psychological, 2 = Integrated, 3 = Medical; Closeness, Dangerousness, Avoidance: 1 = low, 2 = medium, 3 = high. Relevant probability values used for the latent class interpretation are evidenced in bold.*

*Class 2* was the largest class (LCA probability, *p* = 0.74), which included Italy (0.74) as cultural background and where a slight majority of respondents declared to be religious (0.55). Class 2 members were more likely to consider schizophrenic individuals as not particularly dangerous (0.58), but they also expressed a low desire for closeness (0.53) and they would recommend a psychological intervention as the best treatment approach (0.52). A pattern of mixed results emerged for the etiological beliefs, with a similar probability of endorsing psychosocial (0.35), bio-psychosocial (0.30), and biological (0.35) causes. Also avoidance toward individuals suffering from schizophrenia presented a rather undefined pattern, with probabilities 0.31, 0.40, and 0.29 for low, medium, and high levels of avoidance, respectively. Gender of the vignette protagonist did not play any substantial role, with both genders showing a probability value of 0.50.

*Class 1* had a smaller probability than Class 2 (LCA *p* = 0.26) and was primarily defined by Israeli cultural background (0.87) with a predominant religious belief (0.61). Class 1 was primarily characterized by a high level of perceived dangerousness of schizophrenic individuals (0.78), despite a low avoidance (0.77) and medium (0.41) to high (0.46) levels of closeness. Probability values for etiological beliefs and recommended treatment did not suggest a strong tendency: the participants were mostly inclined to endorse bio-psychosocial etiological factors (0.50) followed by psychosocial causes (0.32), and a medical treatment approach (0.41), followed by a combined treatment approach (0.35). Within Class 1, a female protagonist of the vignette had a slightly greater influence (0.54).

A further examination of the identified LCA latent structure involved the analysis of each latent class in relation to the external covariate “cultural background,” which allowed for a relative comparison of the stigma components between the two countries, Italy and Israel.

*Italy* resulted to be better associated with Class 2 of the latent variable (0.74) with a slightly greater proportion of religiosity (0.55). Members of this class are more likely to perceive individuals suffering from schizophrenia as not very dangerous or moderately dangerousness, although socially rejecting both personally and in the public opinion. Further, despite no clear dominance of any type of etiological factors, psychological intervention approaches are indicated as the most eligible.

*Israel* was substantially associated with Class 1 (*p* = 0.87), with a stronger endorsement of religious beliefs (0.61). Members of this class are more likely to perceive people suffering from schizophrenia as very dangerous, although accepting the possibility of moderate and high levels of closeness to them and recognizing that in the public opinion no strong need for avoidance toward schizophrenic people is present. Concerning causal beliefs, the majority of members of this class are likely to endorse a mix of psychosocial and biogenetic etiological factors and, to a smaller extent, exclusively psychosocial, although there is a greater tendency to recommend a medical or integrated treatment approach. Female schizophrenic individuals seem to be slightly more likely to be associated with this class.

### The Strength of Association between the LCA Variables

Latent class analysis results confirmed the consistency of the hypothesized LCA model. Further, the log-linear parameterization of the LCA model allowed us to test the interactions between the latent variable and both the external and the manifest variables, and among the manifest variables. The results of the interaction analysis are presented in **Table 2**.

**Table 2:** Log-linear analysis of the associations between the latent variable and the external variable, the manifest variables, and of the interaction between the manifest variables: *Wald* statistic, degrees of freedom (*df*), and error probability (*p*).

Association between variables	*Wald*	*df*	*p*
Latent variable/Country	34.92	1	<0.0001
Latent variable/Causal beliefs	6.70	2	0.035
Latent variable/Recommended treatment	14.79	2	0.001
Latent variable/Closeness	17.98	2	<0.0001
Latent variable/Dangerousness	5.85	2	0.05
Gender/Dangerousness	10.39	2	0.006
Closeness/Dangerousness	12.55	4	0.014
Closeness/Avoidance	8.86	4	0.05
Dangerousness/Avoidance	11.91	4	0.018

**Table 2** shows that the latent variable identifying the structure of belief and stigmatizing attitudes toward schizophrenic patients is substantially associated with the external variable country and with four of the manifest variables: causal beliefs, recommended treatment, closeness, and dangerousness, which appeared to be good indicators of the latent variable. Contrary to our hypotheses, avoidance resulted to be not associated with the latent variable. More precisely, the association between the latent variable and country confirmed the country-based distinction between the two classes (for both *z* = 5.910, *p* < 0.001). The interaction of causal beliefs with the latent variable showed a significant relation between the latent variable and the level of causal belief that expressed for both classes indecision about the origin of schizophrenia (*z* = 2.43, *p* < 0.01). The interaction between the latent variable and the recommended treatment was represented by a significant positive association of class 1 represented mainly by Italians with psychological treatments (*z* = 3.73, *p* < 0.001) and of class 2 represented by Israelis with medical treatments (*z* = 2.51, *p* < 0.01). The relation of the latent variable with closeness was represented by the associations of class 2 with low closeness (*z* = 4.193, *p* < 0.0001) and of class 1 with high closeness (*z* = 2.991, *p* < 0.01). Latent variable and dangerousness association was not very strong and was mainly due to a tendency to a significant interaction between class 2 and low dangerousness (*z* = 1.64, *p* = 0.05) and similarly of class 1 with high dangerousness.

Not distinguishing the two classes, the interaction between gender of schizophrenic individuals and perception of dangerousness was mainly driven by a significant association between low dangerousness and female schizophrenic persons (*z* = 3.024, *p* < 0.001). Low dangerousness was also related to high closeness (*z* = 3.417, *p* < 0.001), while high dangerousness was strongly associated with a strong perception of avoidance (*z* = 6.40, *p* < 0.001).

## Discussion

The present study tested two main research questions concerning stigmatizing attitudes and beliefs toward schizophrenia: (a) whether the association among etiological beliefs, recommended treatments, social closeness, dangerousness, and avoidance of schizophrenic individuals could be accounted for by a common underlying latent variable and (b) whether different cultures – in this case Italian and Israeli – religious beliefs, and gender of individuals suffering from schizophrenia could be considered both a covariate and a moderator of the same latent variable. Both questions had a positive answer on the base of a hypothetical LCA latent structure representing the relations among such variables. Two main sets of results were found: (A) the reciprocal relevance of the six manifest variables as indicators of the latent variable and (B) the differential effects of the two cultures and religious beliefs on the latent structure, and their relations, allowed to interpret a two-class latent categorical dimension represented by prejudicial attitudes, causal beliefs and treatment options concerning schizophrenia. As known, among other mental disorders, schizophrenia has been extensively studied, so that the results of the present study can find a well-defined context for their interpretation. Studies by Phelan et al. (2006), Schomerus et al. (2011), and by Mannarini and Boffo (2015), where schizophrenia was analyzed in the context of other mental disorders, appeared fundamental to understanding the specificity of that mental problem in relation to causal beliefs, treatments, and stigmatizing attitudes. The LCA allowed to evidence two profiles characteristic of schizophrenia each one corresponding to a latent class, where class 1 was clearly represented by the responses of the Israeli participants with a probability of 0.87 and class 2 was representative of the responses of the Italian participants with a probability of 0.74.

In *class 1* the patient’s gender, in particular the female gender, was considered relevant in the definition of schizophrenia, whereas etiology was uncertain. As far as treatment is concerned, medical treatment was indicated as the best. Concerning stigmatizing attitudes, although dangerousness was considered high, personal closeness to the schizophrenic patient was not excluded, together with the perception of scarce need for avoidance in the public opinion. These results find a well-defined context for interpretation in the literature on mental illness and prejudice in Israel. Mazor et al.’s (2016) research with Israeli participants explained that due to historical and social events, there is a significant presence of high rates of exposure to trauma among mentally ill people. Tal et al.’s (2007) results indicated that although the public manifest negative attitudes toward persons with mental illness, with schizophrenic and depressive symptoms in particular, Israelis do not manifest avoiding attitudes toward them and they are favorable to the activation of rehabilitation programs and all kinds of efforts to promote access and life opportunities of individuals with mental illness.

In *class 2*, Schizophrenia etiology was not well defined and psychological treatments was recommended by the majority for that pathology. The majority of participants considered schizophrenia not very dangerous, and consequently, they perceived low levels of avoidance in the public opinion, but at the same time, they expressed low levels of personal and social desire for closeness. These results confirmed previous finding with Italian participants in the study by Mannarini and Boffo (2014b), where as far as schizophrenia was concerned, social distance and dangerousness seemed to be in contrast: namely low dangerousness interacted with high social distance probably indicating that the reason for refusing schizophrenics is not feeling unsafe, but it is placed somewhere else. Low avoidance, namely not refusing a possible involvement with schizophrenia seemed to be also in contrast with manifesting a desire to be in a relationship with a mentally ill person. An explanation can be found in the definition of the two constructs, where in this study, as far as avoidance is concerned, respondents were asked to manifest their opinion about what in general people think in terms of involvement with a mentally ill person, whereas concerning social distance, the respondents were asked to say if they were willing to be personally in a relationship with the person described in the vignette. The relationship varied in its degree of closeness and the participants were asked if they would accept the person as a close friend, as an acquaintance, as a co-worker, and as a neighbor. The belief about the etiology of schizophrenia was included in this study to better understand the mental illness related to stigmatizing attitudes and recommendation for the best treatment to be undertaken. Both class 1 and class 2, respectively, characteristics of Israelis and Italians, did not offer clear indications of the respondents’ opinions, although Israelis showed a more marked tendency than Italians toward either psychological or psychological/biological causes.

These undefined results demonstrated a scarce knowledge of specific aspects of a mental disorder such as schizophrenia and may be considered one of the reasons for stigmatizing ill people considering them very dangerous as evidenced in class 1 or not desirable in order to establish a close relationship with them as found in class 2. Treatment intervention usually interacts with belief about causes (Mannarini and Boffo, 2015). In this study the two variables did not show an interpretable connection, but within both classes, a rather clear indication emerged as far as treatment is concerned, namely in class 1 mostly represented by Israelis, either a medical or an integrated treatment approach was indicated. This finding is in line with Phelan et al. (2006), which argued that the public opinion recommends for schizophrenia medical treatments and hospitalization but does not recommend searching for other professionals’ help, such as a psychiatrist or a psychotherapist. In class 2 mostly determined by Italian responses a psychological approach was evidenced as related to schizophrenia; this result seemed to confirm previous studies with Italian subjects (Mannarini and Boffo, 2015); more in general, various very recent studies took into consideration the positive effect of specific psychological approaches in the treatment of schizophrenia (Kani et al., 2017; Steel et al., 2017). In summary, in this study the discussion on the plausibility that a clearly oriented knowledge of the etiology of mental illness related to a clear opinion about the best treatment to be undertaken would improve the understanding of mental illness and would limit the people’s prejudice is still open. This general result was in part better specified in the Israeli responses, where although Israelis consider schizophrenia dangerous and they think in the majority that it should be treated medically, they did not express stigmatizing attitudes in terms of personal closeness and avoidance. An explanation could be found in the fact that they consider schizophrenia an illness not being under the voluntary control of the ill person. Italians’ results were rather unspecified: after declaring that schizophrenia is quite dangerous, they did not reject a close relationship with a schizophrenic; they also seemed to prefer psychological treatments to medical ones.

The LCA modeling applied in this study allowed to obtain a structure of the variables under consideration as hypothesized; two interpretable patterns emerged associated with two different clusters of variables describing different aspects of schizophrenia. The modeling of the data by means of the LCA allowed in particular to obtain two well-distinguished classes typical of the two countries the participants belonged to.

Further research is needed to overcome the limitations of this study, first of all the generalizability of the results found on these specific samples. Other samples, different from psychology students, should be investigated, including people with different cultural backgrounds and educational levels in order to generalize the validity of the results. From a cultural perspective, it might be useful to take into account other religions different from Hebrew and Catholicism also. However, as already underlined, the purpose of this study was to compare two levels “being religious” versus “not being religious,” regardless of the type of religion. Also different levels of age should be considered. Furthermore, in order to confirm and deepen the results obtained herein, other schizophrenia stereotypes and prejudices should be included. The value of this study might be improved by analyzing other mental diseases also and comparing them with schizophrenia. From a methodological point of view other approaches such as an implicit measurement should be applied in order to discover other aspects of schizophrenia.

## Author Contributions

SM wrote the manuscript. SM, MB, LB, and AR conceived and designed the study. SM, LB, and AR managed the data collection. SM performed the analysis and SM and MB contributed to data discussion and interpretation. All the authors read, critically revised, and approved the final manuscript and agreed to be accountable for all the aspects of the study.

## Conflict of Interest Statement

The authors declare that the research was conducted in the absence of any commercial or financial relationships that could be construed as a potential conflict of interest.
